# Sit-to-Stand in People with Stroke: Effect of Lower Limb Constraint-Induced Movement Strategies

**DOI:** 10.1155/2014/683681

**Published:** 2014-03-16

**Authors:** Charla Krystine Gray, Elsie Culham

**Affiliations:** School of Rehabilitation Therapy, Queen's University, Kingston, ON, Canada K7L 3N6

## Abstract

*Background*. Weight-bearing asymmetry and impaired balance may contribute to the increased fall risk in people with stroke when rising to stand from sitting. *Objective*. This study investigated the effect of constraint-induced movement (CIM) strategies on weight-bearing symmetry and balance during sit-to-stand in people with stroke. *Methods*. A nonrandom convenience sample of fifteen people with stroke performed the sit-to-stand task using three CIM strategies including a solid or compliant (foam) block strategy, with the unaffected limb placed on the block, and an asymmetrical foot position strategy, with the unaffected limb placed ahead of the affected limb. Duration of the task, affected limb weight-bearing, and centre of pressure and centre of mass displacement were measured in the frontal and sagittal plane. *Results*. Affected limb weight-bearing was increased and frontal plane centre of pressure and centre of mass moved toward the affected limb compared to baseline with all CIM strategies. Centre of mass displacement in the sagittal plane was greater with the compliant block and asymmetrical foot strategies. *Conclusions*. The CIM strategies demonstrated greater loading of the affected limb and movement of the centre of pressure and centre of mass toward the affected limb. The compliant block and asymmetrical foot conditions may challenge sagittal plane balance during sit-to-stand in people with stroke.

## 1. Introduction

People with stroke have a higher risk of falling compared with their age matched peers [1–4] with many falls occurring during transition movements including rising to stand from sitting [1, 5, 6]. STS in people with stroke is characterized by greater loading on the unaffected limb [6–10] and larger frontal plane centre of pressure (COP) displacement compared with age matched healthy adults [8, 10, 11]. Previous authors have equated larger total COP displacement with balance impairment [8, 10, 11]. Weight-bearing asymmetry and impaired balance may contribute to the increased fall risk in people with stroke [8]. Consequently, improved weight-bearing symmetry and balance during STS are goals of rehabilitation in this population.

Constraint-induced movement (CIM) therapy is a treatment strategy designed to increase affected limb weight-bearing during STS in people with stroke. Three CIM strategies for the lower limb include placement of the unaffected limb ahead of the affected limb [7, 9, 12–16] and placement of the unaffected limb on a solid [14] or compliant (foam) [7] block during STS practice. The CIM strategies are designed to increase loading of the affected limb by placing the unaffected limb in a position of biomechanical disadvantage by altering joint angle and length of the hip and knee musculature and in the case of foam, reducing sensory input from the unaffected limb.

Investigations of CIM strategies for the lower limb have primarily focused on the effect on weight-bearing and joint moment asymmetry. STS performed with the unaffected limb placed half a foot length ahead of the affected limb reduced weight-bearing asymmetry [7, 9, 12, 14] and joint moment asymmetry [9, 13] at the knee compared with STS performance with the feet placed in parallel. Rocha et al. [14] reported less weight-bearing asymmetry in people with stroke during STS when the unaffected limb was placed on a solid block, height equal to 25% of seat height. Conversely, Brunt et al. [7] reported no change in weight-bearing asymmetry when the unaffected limb was placed on a compliant block, height equal to 25% of chair height.

There has been little investigation of the effect of CIM strategies on measures reflecting balance, including total COP and COM displacement [15]. Investigation of the effect of CIM strategies on measures of balance is important as training with these strategies may improve balance and reduce the fall risk in people with stroke when performing transfers including the STS task. For example, Cheng et al. [17] demonstrated a smaller frontal plane COP displacement and reduced risk of falling in people with stroke following symmetrical weight-distribution training during STS. Duclos et al. [15] reported less frontal plane COP displacement in people with stroke when they performed the STS task with the unaffected limb placed half a foot length ahead of the affected limb, compared with the feet placed in parallel [15]. No studies have reported the effect of CIM strategies on postural control in the sagittal plane or in the frontal plane with the solid or compliant blocks.

Given the complexity of the STS task [18], people with stroke may find it difficult to rise to stand from sitting with the CIM parameters previously investigated. The effect of placing the unaffected limb only a quarter of a foot length ahead of the affected limb or placement of the unaffected limb on a block height less than 25% of the chair height on STS performance in people with stroke has not been investigated. It is possible that lower block heights and a smaller asymmetrical foot position offset are also effective in increasing affected limb loading and improving balance.

The purpose of this study was to investigate the effect of different levels of three CIM strategies for the lower limb on temporal, weight-bearing, and COP and center of mass (COM) displacement measures of STS performance in people with stroke. We hypothesize that solid block heights less than 25% of the chair height, placement of the unaffected limb less than a half-foot length ahead of the affected limb, and the compliant blocks will increase loading of the affected limb and centralize the position of the COP and COM in the frontal plane at seat-off. We hypothesize that the lower density compliant block will have a greater effect than the higher density as it is a less stable surface. Lastly we hypothesize that the strategies will not increase frontal plane instability as reflected by COP and COM displacement during STS.

## 2. Methods

### 2.1. Participants

Participants were included if it was their first known stroke, at least six months since stroke onset, only one side of the body was affected by the stroke, and they were able to rise from a chair without using their arms. Exclusion criteria included a known history of neurological impairment other than stroke affecting the lower limbs and neglect of space on the affected side. The university research ethics board approved the study protocol and all participants provided informed consent.

### 2.2. Instrumentation

Kinematic data were collected at 50 Hz with an OPTOTRAK 3020 system (Northern Digital Inc., Waterloo, Canada). Three AMTI (AMTI, Newton, and MA) force platforms (FPs) were used to gather kinetic data with a sampling frequency of 100 Hz. One force platform (FP) was placed under the chair and one FP was placed under each foot. Clusters of three or four infrared light emitting diodes (IREDs) mounted on rigid plastic molds were placed on the 7th cervical vertebra, sacrum, and bilaterally on top of the foot, lateral midshank, and lateral midthigh. At the end of the STS trials, participants stood in view of both cameras for collection of a series of reference trials used to identify landmarks in relation to the IRED clusters. Landmarks were identified bilaterally with the tip of a probe and included the first and fifth metatarsal heads, lateral and medial malleoli, lateral and medial femoral epicondyles, greater trochanter, a point aligned vertically with each greater trochanter at the level of the anterior superior iliac spine, and the acromion process of the scapula. The probe was instrumented with four IREDs in a fixed orientation to the tip. The landmark data combined with the marker and anthropometric data were used to create an eight-segment model in C-Motion (Visual 3D, C-Motion Inc., Germantown, MD) for calculation of total body COM and to identify the end of the STS task.

Kinematic and kinetic data were filtered (lowpass, 6 Hz Butterworth) and synchronized using visual 3D motion analysis software (visual 3D, C-Motion Inc., Germantown, MD). An eight-segment model was created including bilateral foot, shank, and thigh, the pelvis, and trunk. Segment lengths and joint centers were calculated based on the position of the landmarks (reference trials) in relation to the IRED clusters. A combined vertical ground reaction force (VGRF) was calculated in C-Motion by adding the VGRF value from the FP under each foot. A net frontal and sagittal COP path was calculated in C-Motion using an equation from Winter et al. [19].

### 2.3. Protocol

Participants attended a single test session in a university motion analysis research laboratory. Demographic data, including age, height, weight, and time, since stroke onset were collected prior to the start of the test session for descriptive purposes and to ensure that participants met the inclusion criteria.

With IREDs in place, participants were asked to perform a series of STS trials from an armless, backless, and height adjustable chair, wearing a pair of comfortable shoes. Chair height was standardized to the height of the participant's knee (distance between the knee joint line and the floor). Participants sat with one-third of their thigh length on the chair (distance between the greater trochanter and the lateral knee joint line). Participants were instructed to perform the STS task at a self-paced speed with their arms folded across their chest while looking at a target located 1.6 meters above the floor surface. Tape was placed on the FPs to ensure consistency of foot placement between trials and stickers were placed on the chair and the participant's thighs to promote consistency of seat position between trials. Three seconds of quiet sitting were collected at the beginning of each trial to provide a baseline measure of the VGRF under the chair.

Participants performed the STS task with no CIM strategy (baseline) and with three CIM strategies (solid block (SB), compliant block (CB), and asymmetrical foot position strategies). For all conditions, the medial border of the heels was placed 10–15 cm apart, as per participant comfort and the baseline sit-to-stand. Solid and compliant block conditions were performed with the feet placed in parallel with the unaffected limb placed on the block. Four solid block heights were tested, 2.54 (*SB1*), 5.08 (*SB2*), 7.62 (*SB3*), and 10.16 cm (*SB4*). The CBs were 10.16 cm in height and constructed from Rebond foam. Two CB densities were tested, 2.72 kg (*CB1*) and 6.63 kg (*CB2*). The asymmetrical foot condition was performed with the unaffected foot placed a quarter (*quart*) or a half (*half*) a foot length ahead of the affected foot; foot length was measured as the distance between the distal end of the first toe and the most posterior aspect of the heel. Participants performed three baseline STS trials and three consecutive trials of each condition with one practice trial of each condition prior to collecting data for analysis. The baseline condition was always performed first followed by random presentation of each CIM strategy. Randomization was performed prior to each test session by pulling pieces of paper with the name of each condition (*SB1, SB2, SB3, SB4, CB1, CB2, quart, or half*) out of a hat. To minimize possible carry-over effects between conditions, two solid block, compliant block, or asymmetrical foot conditions were not presented consecutively.

### 2.4. Outcome Measures

*Time*. Time to complete the STS cycle was defined, in seconds, from the start of task (first change in the VGRF under the chair from the baseline values) to the end of the task (hip reached full extension).

*Weight-Bearing*. Affected limb VGRF at seat-off (WB) was expressed as a percent of the total VGRF under the two feet at seat-off. Seat-off was defined as the first frame when the VGRF under the chair reached zero.

*Frontal Plane Displacement*. Frontal plane displacement of the center of pressure (COP_x_) and center of mass (COM_x_) in centimeters was measured as the distance between the most right and left lateral positions of the COP and COM between the start and end of the STS cycle. The distance between the COP and COM position and midline (midpoint between the medial malleoli) were measured in cm (COP_x__midline and COM_x__midline) at seat-off.

*Sagittal Plane Displacement.* Sagittal plane center of pressure (COP_y_) and center of mass (COM_y_) displacement, in centimeters, were measured as the distance between the most anterior and posterior positions of the COP and COM between the start and end of the STS cycle. The distance between the COP and COM position and the midpoint between the medial malleoli in the sagittal plane at seat-off were measured in centimeters (COP_y__midline and COM_y__midline).

### 2.5. Data Analysis

Descriptive statistics (mean and one standard deviation) were calculated for each variable across all conditions using the average of three trials from each individual. Results for each dependent variable were tested with repeated measures analysis of variance (ANOVA) calculated using IBM SPSS Statistics (version 20). An alpha level of 0.05 was chosen for all comparisons. All data were examined for violation of sphericity, determined by a significant value for Mauchly's test of sphericity. The Greenhouse-Geisser correction was used to determine significance when sphericity was violated. When a main effect of condition was found a post hoc, pairwise comparison with no adjustment (Tukey LSD) was performed to determine the source of the main effect.

## 3. Results

*Participants.* Fifteen people with stroke were recruited from the community. All were independent ambulators in the community, were able to walk into the research laboratory for testing, and ascend/descend stairs (observed entering and leaving the building). Several participants required the use of a handrail when ascending/descending the stairs and only one participant used a cane for ambulation. One participant (CVA07) wore an ankle foot orthosis that was worn during testing and another participant (CVA06) had a knee joint replacement of the unaffected limb five years prior to the study. Participant characteristics are presented in Table 1. Mean values for all outcome measures across all conditions are provided in Table 2.

Thirteen participants were able to perform the STS task across all conditions. The mean age, height, weight, and time since stroke onset of these thirteen participants were 66.7 ± 11.8 years, 1.7 ± 0.1 m, 83.8 ± 10.1 kg, and 33.1 ± 32.5 months, respectively. All thirteen participants safely performed the STS task with the CIM strategies. None of the participants demonstrated unsteadiness during testing that required assistance to regain balance. One participant (CVA09) was only able to perform the STS task with the baseline and SB1 conditions and a second participant (CVA07) was only able to perform the STS task with the baseline, SB1, SB2, and the quart-foot conditions. These two participants were not able to rise without arm use in the remaining conditions. Statistical analysis was carried out on the 13 participants who were able to perform the STS task across all conditions. Statistical analysis for COM measures was carried out on 12 participants due to instrumentation error for one participant.

The assumption of sphericity was violated for the following measures: time, COP_x_, COM_x_, COP_y_, COM_y_, and COP_y__midpoint. For all of these measures, the Greenhouse-Geisser correction was used to determine a main effect of condition.

*Time.* Addition of the CIM strategies did not affect the time needed to complete the STS task (*P* > 0.05, Table 2). Average time to complete the task varied from 2.9 (SB3) to 3.1 seconds (half-foot). Observation of the raw data for the two subjects unable to complete the task across all strategies suggested no change in time needed to complete the task with the strategies that were successfully completed.

*Weight-Bearing. *There was a main effect of strategy on affected limb weight-bearing (WB) at seat-off (*P* < 0.001) (Table 2). All CIM strategies resulted in a significant increase in affected limb WB compared to baseline. Average affected limb WB with the half-foot strategy was 55.1 ± 7.2%, which was significantly higher than all other CIM strategies.

Observation of the raw data for subject CVA07, with the AFO, suggested greater loading of the affected limb with the conditions completed successfully (SB1, SB2, and quart foot). There was no change in affected limb loading for subject CVA09 with the SB1 condition. 

*Postural Control in the Frontal Plane.* CIM strategies did not affect the total displacement of either COP or COM in the frontal plane during the STS task (*P* > 0.05, Table 2). All CIM strategies resulted in a shift of the COP position at seat-off toward the affected limb (*P* < 0.001). The mean COP position was on the unaffected limb side of midline with the baseline, SB, and CB strategies and toward the affected limb relative to midline with the quart-foot and half-foot strategies (Table 2). Pairwise comparison revealed that the shift with the half-foot condition was greater than for all other CIM strategies.

There was also a main effect of strategy for the COM position at seat-off, with a shift of the COM toward the affected limb (COM_x__midline) (*P* < 0.001). Pairwise comparison revealed that all strategies, except SB2, resulted in a COM position closer to the affected limb compared to baseline. The COM moved toward midline with the SB and CB conditions. With the quart-foot and half-foot conditions, the COM was located toward the affected limb relative to the midline (Table 2). The shift with the half-foot condition was greater than for all other strategies.

Observation of the raw data for subjects CVA07 and CVA09 suggested no change in measures of frontal plane balance with the strategies that were successfully completed.

*Postural Control in the Sagittal Plane.* Addition of the CIM strategies did not affect total displacement of the COP in the sagittal plane (COP_y_) (*P* > 0.05). There was a main effect of strategy for COM displacement (COM_y_) (*P* < 0.001). The COM moved further forward with the CB, quart-foot, and half-foot strategies compared with baseline. Pairwise comparison revealed a significantly greater forward movement with the half-foot strategy compared with all other strategies (Table 2).

There was a main effect of strategy for COP and COM position at seat-off (COP_y__midpoint and COM_y__midpoint) (*P* < 0.001). With the compliant block strategies, the COP and COM position were further forward compared with baseline (Table 2) whereas with the asymmetrical foot conditions the COP and COM position were further posterior compared with baseline (Table 2).

Observation of the raw data for subject CVA07 demonstrated less total excursion of the COP and COM with the conditions successfully completed and no change in the sagittal plane COP and COM position at seat-off. There was no change in measures of sagittal plane balance for subject CVA09 with the SB1 condition.

## 4. Discussion

None of the CIM strategies investigated altered time to complete the task, total frontal plane COP and COM displacement, or total sagittal plane COP displacement. There was a significant increase in affected limb loading at seat-off and a shift in the frontal plane COP and COM (except SB2) toward the affected limb at seat-off with all strategies. Total sagittal plane COM displacement was greater with the compliant block and asymmetrical foot position conditions compared to baseline. In the sagittal plane, the COP and COM were positioned more anteriorly at seat-off with the compliant block conditions and more posteriorly with the asymmetrical foot position conditions compared to baseline.

Two participants in this study were unable to stand from sitting without using their arms with some of the strategies. This appeared to be due to insufficient muscle strength. They may have been unable to generate sufficient muscle force with their unaffected limb placed in a position of biomechanical disadvantage.

Although the time to complete STS was highest with the quarter- and half-foot strategies, none of the strategies significantly increased the time for STS compared to the baseline condition. This is contrary to findings by Camargos et al. [16] who reported that people with stroke required more time to rise to stand with an asymmetrical foot position compared to STS with the feet placed in parallel [16]. This difference may suggest a higher level of function in people with stroke in the current study compared with Camargos et al. [16]. In the present study all participants were able to walk into the research laboratory for testing, ascend/descend stairs (observed entering and leaving the building), and rise to stand without using their arms. The difference between studies may also reflect methodological variation for performing the STS task. In both studies, the chair height equaled 100% of the participant's knee height but the knee and ankle joint angles may have differed between studies.

All strategies significantly increased weight-bearing on the affected limb as hypothesized. The magnitude of the increase in affected limb weight-bearing was not dependent on the density of the foam block, contrary to our expectations. The half-foot strategy resulted in affected limb weight-bearing greater than 50% of the total weight-bearing. With all other strategies, affected limb weight-bearing approached symmetry with the unaffected limb, suggesting the greatest forced-use of the affected limb with the half-foot strategy.

Greater affected limb loading with CIM strategies is consistent with results previously reported with a solid block placed under the unaffected limb [14] and with an asymmetrical foot strategy [7, 9, 12]. Rocha et al. [14] reported greater loading of the affected limb (45% of total limb load), when a solid block, equal to 25% of the chair height, was placed under the unaffected limb compared with both feet placed in parallel and on the floor (37.5% of total limb load). The highest solid block (SB4) was similar in height to the block used by Rocha et al. [14] and resulted in greater affected limb loading compared to the three lower block heights. Findings from this study demonstrated greater affected limb loading with lower block heights and, therefore, lower blocks could be used clinically in people with stroke with greater sensorimotor impairment who are unable to perform the task with the higher block height. This is also suggested by the greater affected limb loading with the SB1 and SB2 condition for CVA07.

Affected limb loading increased significantly with both compliant blocks. Conversely, Brunt et al. [7] reported no effect of foam on affected limb loading compared to STS with feet in parallel and on the floor (baseline). The difference between studies may be explained by the willingness of participants to accept greater loading of the affected limb in the current study when performing STS with the compliant blocks possibly reflecting greater muscle strength, sensory awareness, or balance confidence. Contrary to our hypothesis there was no difference in the magnitude of affected limb loading with the two block densities suggesting that the block height and sensory manipulation affect limb loading rather than foam density.

Findings from previous studies investigating STS with the asymmetrical foot strategy reported values approaching symmetry of loading between the two limbs [7, 9, 12]. Lecours et al. [9] reported an affected to unaffected limb ratio of 0.66 ± 0.24 with the feet placed in parallel and 0.87 ± 0.32 with the unaffected limb placed a half-foot length ahead of the affected limb. These ratios reflect an affected limb loading equal to 40% and 46% of the total limb load, respectively. In the current study limb loading values approached symmetry with the quart-foot condition and affected limb loading was greater than unaffected limb loading with the half-foot condition. In the present study, participants may have been willing to accept greater loading of the affected limb due to greater muscle strength, sensory awareness, or balance confidence. The difference in affected limb loading between studies may also reflect the inherent variability of participant response within a clinical population. Findings from this study demonstrated that an asymmetrical foot position less than a half-foot length offset resulted in greater affected limb loading compared to baseline and could be used clinically with people with stroke who are unable to stand with the half-foot length offset.

In the early (less than six months) stages of recovery following a stroke, learned nonuse of the affected limb may develop when attempts to perform the STS task with equal limb loading are unsuccessful [20]. With repeated unsuccessful attempts, a compensatory pattern for performing the STS task with greater loading on the unaffected limb may be reinforced [20]. Limb loading asymmetry may also develop during recovery to reduce the risk of falling toward the affected limb by compensating for muscle weakness and impaired sensory awareness of the affected limb. Benefits of training STS with greater loading on the affected limb may include increased muscle strength and increased confidence placing weight through the affected limb by maximizing joint compression and augmenting sensory awareness of the limb. Increased muscle strength and confidence placing weight through the affected limb may reduce the fall risk during STS. Another potential benefit is that greater attention to the affected limb may reverse the effects of learned nonuse. Greater use of the affected limb with the CIM strategies may provide a functional method for training muscle strength and joint position sense and may reverse the effect of learned nonuse [20].

All CIM strategies resulted in a shift of the COP toward the affected limb and all strategies (except SB2) resulted in a shift of the COM toward the affected limb at seat-off. These findings are consistent with Duclos et al. [15] who reported less COP deviation from midline between 35 and 80% of the STS task with an asymmetrical foot strategy (1 cm from midline toward unaffected limb) compared with the feet placed in parallel (3 cm from midline). The centralized COP and COM position promote symmetry of movement at seat-off and may reduce the risk of falling.

People with stroke have demonstrated greater COP and COM displacement in the frontal plane compared with healthy adults during STS and this quantified measure of balance may contribute to a higher risk of falling in people with stroke [8]. Greater weight-bearing asymmetry has been associated with a larger COP displacement and an increased risk of falling [8]. Although the COP and COM moved closer to midline and greater loading of the affected limb was observed with the CIM strategies, there was no change in the total frontal plane COP and COM displacement with the CIM strategies. This finding suggests that factors other than weight-bearing contribute to COP and COM displacement and frontal plane balance mechanisms. This might include strength of the hip abductor and adductor muscles, which control trunk position in this plane [19]. Although the CIM strategies did not affect total frontal plane COP and COM displacement in a single session it is possible that training using these strategies may lead to improved postural control in the frontal plane over time as demonstrated by Cheng et al. [17].

There was no change in total sagittal plane COP displacement with the strategies. The compliant block strategies resulted in greater sagittal plane COM displacement, compared to baseline and a more anterior position of the COP and COM at seat-off. The larger COM displacement with the compliant block strategies suggests a challenge to the sagittal plane postural control mechanisms when using this strategy. The more anterior position of the COM at the critical point of seat-off may be necessary to minimize forward movement of the COM and the number of postural corrections required following seat-off. Hesse et al. [21] reported that with the feet placed in parallel, the centre of gravity is further forward over the base of support in people with stroke compared with healthy adults and argued that this was a more favorable position for the transition from the three-point to two-point base of support. This was also demonstrated in people with Parkinson's disease and it was suggested that it is a strategy to redistribute the lower extremity joint moments to compensate for an inability to generate lower extremity muscle force [22]. The height of the two compliant blocks was the same as the highest solid block. COM displacement and COP and COM position at seat-off were not altered with the highest block strategy, suggesting that findings with the compliant block strategies were likely due to the altered sensory information rather than the height of the block.

Sagittal plane COM displacement was larger and the COP and COM position were further posterior with the asymmetrical foot strategies. These findings are most likely related to the change in the base of support (BoS) in the sagittal plane with these strategies. The COM must move further forward during the STS task due to the increase in the length of the BoS in order to bear weight on the more forward limb. The change in BoS likely also explains the more posterior position of the COP and COM at seat-off as more of the weight is taken on the more posteriorly placed limb.

Previously investigated methods of altering STS performance in people with stroke include repetitive task practice (RTP) alone [23], RTP with limb loading feedback [17], or RTP with altered knee joint angles and altered floor surface [4]. Repetitive task practice (RTP) with these strategies resulted in a faster time to rise to stand from sitting [4, 17], greater loading of the affected limb [17, 23], and a smaller frontal plane total COP displacement [17]. Repetitive task practice with one of the CIM strategies may also result in improved STS performance following training. Further research is needed to compare strategies to increase affected limb loading to determine which is most effective.

A strength of this study is the ability to compare the effect of three CIM strategies. All three CIM strategies resulted in greater affected limb loading and centralization of the frontal plane COP and COM position at seat-off. Only the compliant block and asymmetrical foot position conditions altered sagittal plane measures of postural control. Another strength of this study is that it demonstrated that all block heights and both asymmetrical foot positions resulted in greater affected limb loading and movement of the COP and COM toward the affected limb. Therefore, participants could start with a lower level of the condition and progress to a higher level when able. These findings provide justification for a randomized controlled trial (RCT) utilizing the CIM strategies in a RTP paradigm to determine their effect on STS performance, carry-over of any effects to gait, and standing balance and retention of any effect six months after training compared to STS practice without the use of CIM strategies.

Findings from this study are limited to people with stroke who are able to walk independently, ascend/descend stairs, and rise to stand without using their arms. All conditions aimed to increase affected limb loading. Therefore, it is possible that there was a carry-over effect in that, once forced to increase load on the affected limb, participants may have been more willing to do so in subsequent trials. The order of the conditions was randomized and there was no indication that this occurred on review of individual subject data. Limb loading varied across conditions and was not dependent on order of presentation.

## 5. Conclusions

Performing STS with all of the CIM strategies increased affected limb loading and resulted in a significant shift in the frontal plane COP and COM position (except SB2) toward midline at seat-off. Sagittal plane COM displacement was increased with the compliant block and asymmetrical foot placement strategies. At seat-off, the COP and COM position were more anterior relative to the BoS with the compliant block conditions and more posterior with the asymmetrical foot position conditions. All strategies may be effective in increasing affected limb loading and centralizing COM over the BoS at seat-off. The asymmetrical foot position strategy is recommended for use in a RCT due to the effect of this strategy on measures of frontal and sagittal plane balance as well as the ability to progress participants from using a smaller asymmetrical foot position off-set (quarter foot length) to a larger foot position off-set (half foot length). The compliant block strategy may provide additional challenges to balance and is also recommended for further study in a RCT.

## Figures and Tables

**Table 1:** Participant characteristics.

Participant	Age (y)	Sex	Weight (kg)	Affected limb	Time since stroke onset (m)
CVA01	68	M	94	R	41
CVA02	55	M	85	R	40
CVA03	64	M	78	L	132
CVA04	52	M	84	L	8
CVA05	46	M	85	R	23
CVA06^†^	72	M	82	R	16
CVA07^∗‡^	64	F	70	R	408
CVA08	66	M	98	L	20
CVA09*	59	M	50	L	18
CVA10	62	M	101	R	7
CVA11	77	F	88	R	18
CVA12	60	M	82	R	25
CVA13	80	F	69	L	53
CVA14	85	M	67	R	23
CVA15	80	F	76	R	24

y: years, M: male, F: female, kg: kilograms, R: right, L: left, and m: months. *Participant unable to complete sit-to-stand with all conditions; therefore data were not included in the analysis; ^†^participant with a knee joint replacement; ^‡^participant wore an ankle foot orthosis.

**Table 2:** Mean (SD) of all measures across all conditions.

Measure	Baseline	SB1	SB2	SB3	SB4	CB1	CB2	Quart	Half
Time (sec)	2.9 (0.6)	2.9 (0.6)	2.9 (0.6)	2.9 (0.5)	3.0 (0.5)	3.0 (0.6)	3.0 (0.6)	3.0 (0.6)	3.1 (0.7)
WB (% total)	45.8 (5.0)	48.4 (6.5)	49.5 (5.6)	49.9 (6.1)	51.6 (5.9)	51.0 (6.8)	50.7 (6.9)	49.9 (7.0)	55.1 (7.2)
COP_x_ (cm)	7.4 (3.9)	5.6 (1.5)	6.1 (2.8)	6.7 (3.2)	7.4 (3.9)	7.2 (3.2)	6.7 (3.0)	7.0 (2.1)	8.6 (2.2)
COM_x_ (cm)*	2.4 (0.8)	2.4 (1.0)	2.8 (0.8)	2.7 (1.0)	3.1 (1.4)	3.1 (0.9)	3.0 (1.2)	2.7 (0.9)	3.0 (1.3)
COP_x__mid (cm)*	1.0 (1.3)	0.4 (1.6)	0.3 (1.3)	0.3 (1.4)	0.0 (1.5)	0.2 (1.5)	0.3 (1.7)	−0.2 (1.8)	−1.8 (1.7)
COM_x__mid (cm)*	0.8 (1.1)	0.3 (1.2)	0.4 (1.3)	0.1 (1.1)	0.0 (1.1)	0.3 (1.3)	0.2 (1.3)	−0.1 (1.1)	−1.2 (1.2)
COP_y_ (cm)	8.7 (2.4)	8.6 (2.2)	9.9 (5.2)	8.3 (2.0)	8.7 (2.0)	7.7 (1.8)	8.6 (1.7)	9.4 (2.4)	10.5 (2.7)
COM_y_ (cm)*	25.4 (2.1)	26.1 (2.7)	26.2 (2.8)	26.1 (2.2)	25.7 (3.3)	26.9 (2.5)	26.7 (2.4)	29.3 (3.3)	32.5 (4.0)
COP_y__mid (cm)*	0.5 (2.2)	0.6 (2.2)	0.4 (2.9)	1.1 (2.6)	0.8 (2.6)	1.9 (2.2)	1.6 (2.4)	−0.2 (2.4)	−0.9 (3.0)
COM_y__mid (cm)*	−7.3 (2.7)	−7.1 (2.8)	−6.9 (2.8)	−7.5 (3.0)	−7.4 (2.9)	−6.5 (3.0)	−6.2 (3.3)	−8.6 (2.7)	−10.4 (3.6)

*n* = 13, *n = 12.

Time, WB: affected limb weight-bearing at seat-off; COP_x_ and COM_x_: COP and COM displacement in the frontal plane, respectively; COP_x__mid and COM_x__mid: distance between the COP and COM position and the midline between the malleoli in the frontal plane at seat-off, respectively; positive and negative values reflect a position toward the unaffected and affected limb relative to midline, respectively; COP_y_ and COM_y_: COP and COM displacement in the sagittal plane, respectively; COP_y__mid and COM_y__mid: distance between the COP and COM position relative to the midpoint between the malleoli in the sagittal plane at seat-off, respectively; positive and negative values reflect a position anterior and posterior to the midpoint, respectively. SB1, SB2, SB3, and SB4 = 2.54, 5.08, 7.62, and 10.16 cm solid blocks, respectively. CB1 and CB2 = 2.72 and 6.63 kg compliant blocks, respectively. Quart and half: quart-foot and half-foot asymmetrical conditions.
